# In Silico Analysis of Non-Conventional Oxidative Stress-Related Enzymes and Their Potential Relationship with Carcinogenesis

**DOI:** 10.3390/antiox13111279

**Published:** 2024-10-23

**Authors:** Fábio Rodrigues Ferreira Seiva, Maria Luisa Gonçalves Agneis, Matheus Ribas de Almeida, Wesley Ladeira Caputo, Milena Cremer de Souza, Karoliny Alves das Neves, Érika Novais Oliveira, Luis Antônio Justulin, Luiz Gustavo de Almeida Chuffa

**Affiliations:** 1 Department of Chemical and Biological Sciences, São Paulo State University (UNESP), Institute of Bioscience, Botucatu 18610-034, SP, Brazil; maria.agneis@unesp.br (M.L.G.A.); matheus.ribas-almeida@unesp.br (M.R.d.A.); wesley.l.caputo@unesp.br (W.L.C.); karoliny.neves@unesp.br (K.A.d.N.); erika.novais@unesp.br (É.N.O.); 2 Biological Science Center, North of Paraná State University (UENP), Bandeirantes 86360-000, PR, Brazil; milena.cremer@uenp.edu.br; 3 Department of Structural and Functional Biology, São Paulo State University (UNESP), Institute of Bioscience, Botucatu 18610-034, SP, Brazil; l.justulin@unesp.br (L.A.J.J.); luiz-gustavo.chuffa@unesp.br (L.G.d.A.C.)

**Keywords:** enzymatic system, cancer, tumor biology, bioinformatic, in silico analysis

## Abstract

Carcinogenesis is driven by complex molecular events, often involving key enzymes that regulate oxidative stress (OS). While classical enzymes such as SOD, catalase, and GPx have been extensively studied, other, non-classical oxidative stress-related enzymes (OSRE) may play critical roles in cancer progression. We aimed to explore the role of OSRE involved in an OS scenario and to assess their potential contribution to carcinogenesis in some of the most prevalent cancer types. Through data mining and bioinformatic analysis of gene and protein expression and mutation data, we identified OSRE with altered expression and mutations across cancer types. Functional pathways involving EGFR, MT-ND, GST, PLCG2, PRDX6, SRC, and JAK2 were investigated. Our findings reveal that enzymes traditionally considered peripheral to OS play significant roles in tumor progression. Those OSRE may contribute to cancer initiation and progression, as well as be involved with cancer hallmarks, such as EMT and invasion, proliferation, and ROS production. In addition, enzymes like SRC and JAK2 were found to have dual roles in both promoting ROS generation and being modulated by OS. OSRE also interact with key oncogenic signaling pathways, including Wnt/β-catenin and JAK2/STAT3, linking them to cancer aggressiveness and therapeutic resistance. Future research should focus on translating these findings into clinical applications, including the development of novel inhibitors or drugs targeting these non-classical enzymes.

## 1. Introduction

Antioxidant enzymes are crucial in reducing oxidative stress (OS) by neutralizing reactive species such as reactive oxygen species (ROS) and reactive nitrogen species (RNS). Among the most studied enzymes are superoxide dismutase (SOD), catalase, and glutathione peroxidase (GPx). SOD catalyzes the conversion of superoxide radicals into oxygen and hydrogen peroxide, which catalase then breaks down into water and oxygen. GPx, a key player in the glutathione (GSH)-dependent system, is involved in the reduction of hydrogen peroxide and organic hydroperoxides. Other enzymatic systems, encoded by genes such as GSR, GSTM1, NOX, DUOX, GCLM, GCLC, NQO1, HMOX1, DHODH, TXN, and PRDX1, though less frequently studied, also contribute to both the generation and regulation of oxidative disturbances [1,2]. The transcription factors NRF2 and KEAP1, central to the NRF2-KEAP1 pathway, regulate many OS-related events. NRF2 binds to antioxidant response elements (ARE) and promotes the transcription of antioxidant defense genes [3].

Mitochondria, highly dynamic organelles, are involved in almost every cellular process, including both the physiological and pathological production of ROS. This mainly occurs during the electron transport chain (ETC) and oxidative phosphorylation (OXPHOS). ETC and OXPHOS rely on the proper functioning of four enzymatic complexes: complex I (NADH-ubiquinone oxidoreductase), complex II (succinate-ubiquinone oxidoreductase), complex III (ubiquinol-cytochrome c reductase), and complex IV (cytochrome c oxidase). The mitochondrial genome encodes 13 polypeptides involved in the ETC, and mutations in mitochondrial DNA (mtDNA) are known to contribute to cancer initiation and progression [4].

Inadequate antioxidant defenses often lead to OS, which plays a significant role in carcinogenesis. Chronic OS results in DNA damage, mutations, and genomic instability, key factors in cancer development [5]. Additionally, OS can activate oncogenic and inflammatory signaling pathways, driving cell proliferation and survival while impairing tumor suppressor proteins and increasing the metastatic potential of cancer cells [6]. Understanding the complex redox dynamics within cancer cells, alongside the roles of various antioxidant enzymes, may be valuable for cancer prevention and therapy.

Despite the diverse risk factors, genetic predispositions, and treatment approaches for different cancers, OS and antioxidant defenses consistently influence tumor initiation, progression, and metastasis [7]. Cancer remains a leading cause of death worldwide, with breast, colorectal, liver, lung, and prostate cancers being the most prevalent [8]. Lung cancer, the most common type globally, is strongly associated with OS due to exposure to cigarette smoke, environmental pollutants, and other carcinogens that generate high ROS levels [9]. Similarly, breast cancer, the most frequent cancer among women, is linked to OS, as estrogen metabolism produces ROS, leading to oxidative damage in DNA and other cellular components [10]. Colorectal cancer, a major cause of cancer-related deaths, is influenced by diet, microbiota changes, and infections like H. pylori, all of which can increase ROS levels in the gastrointestinal tract and promote chronic inflammation [11]. Prostate cancer, the most common malignancy among men, is also affected by OS, with factors such as aging, hormonal changes, chronic inflammation, and exposure to xenobiotics contributing to ROS generation [12]. Liver cancer, particularly hepatocellular carcinoma (HCC), is closely tied to chronic inflammation and OS, which disrupt liver metabolism and various cellular pathways [13]. Notably, OS contributes to resistance to chemotherapy, radiation, and immunotherapy, complicating cancer treatment [14].

Bioinformatics tools and databases are valuable resources for exploring the complex relationships between OS-related genes and proteins in cancer. Platforms such as CBioPortal [15] offer extensive genomic and transcriptomic data from various cancer types, enabling large-scale analyses to uncover molecular mechanisms underlying cancer. Other bioinformatics tools facilitate differential gene expression analyses, identifying genes involved in OS that are differentially regulated in cancerous versus normal tissues. By integrating data from various platforms, recent studies have highlighted genetic alterations that affect susceptibility to oxidative damage and cancer progression, uncovering key processes and molecular functions influenced by OS in different tumors [9,16,17,18,19,20]. Such efforts help map the molecular landscape of cancer, aiding in the discovery of novel biomarkers and therapeutic targets for OS-related cancers.

Given the importance of understanding how OS influences cancer, this study aimed to identify “non-classic” antioxidant enzymes that are deregulated in the most common types of cancer. To achieve this, various bioinformatics strategies were employed.

## 2. Materials and Methods

### 2.1. Data Mining and Processing

The initial step in our analysis was to identify genes associated with oxidative stress-related processes. We accessed the Gene Ontology Resource database [21] (https://geneontology.org/, accessed on 15 April 2024), which offers a structured framework to describe the activities and functions of gene products. To search for relevant proteins, we used the following descriptors: “Reactive Oxygen Species”, “Reactive Nitrogen Species”, “Oxidative Stress”, and “Antioxidant”. Additionally, we included genes involved in the Gene Ontology Biological Processes “GOBP RESPONSE TO OXIDATIVE STRESS” pathway from the Molecular Signatures Database (MSigDB). The resulting gene set was further filtered using R Studio packages, including hgnc [22], biomaRt [23], and org.Hs.eg.db [24], to ensure comprehensive annotation. These packages allowed us to retain only those genes with identifiers such as HGNC, Ensembl gene ID version, ENTREZ, UNIPROT, and enzyme_id. Moreover, we restricted the selection to genes from Homo sapiens species.

### 2.2. Protein–Protein Interaction (PPI) Network and Hub Gene Identification

We performed the analysis of protein interactions using the STRING database (http://string-db.org, accessed on 22 May 2024). By setting the species to Homo sapiens and adjusting the confidence score to the highest level (0.900) to minimize false positives, we constructed a PPI network that excluded any unconnected nodes. The resulting network was visualized and further analyzed using Cytoscape software version 3.10.2, where the CytoHubba plugin was employed to identify hub genes. Validation of these genes in selected cancer types was conducted using ProteomicsDB 2020 [25]. To perform gene family analysis, we utilized the ToppFun module of the ToppGene database (https://toppgene.cchmc.org/enrichment.jsp, accessed on 6 June 2024).

### 2.3. Differential Gene Expression Analysis

For comparisons of gene expression between tumor and normal tissues, we used the Gene Expression Profiling Interactive Analysis (GEPIA2, http://gepia2.cancer-pku.cn/, accessed on 12 June 2024). The significance threshold was set using ANOVA with an adjusted *p*-value < 0.01 and an absolute log fold change (LogFC) of 1.0. Abbreviations followed database standards: Breast Cancer (BRCA), Colon Adenocarcinoma (COAD), Liver Hepatocellular Carcinoma (LIHC), Lung Adenocarcinoma (LUAD), and Prostate Cancer (PRAD). We also used X2Kweb and TRRUST databases to identify potential transcription factors. The multiMiR library was applied to explore microRNAs (miRNAs) that regulate the identified genes, considering only those validated by luciferase reporter assays or qRT-PCR methodologies.

### 2.4. Differential Protein Expression Analysis

To assess protein expression patterns in both healthy and tumor samples, we utilized the Human Protein Atlas (HPA, https://www.proteinatlas.org/, accessed on 5 August 2024). This resource integrates data from antibody-based imaging, mass spectrometry, transcriptomics, and systems biology, providing a comprehensive view of protein expression across various cell types, tissues, and organs. We utilized the R package HPAanalyze [26] to retrieve data. The following parameters were employed: for hpaVisTissue, the target tissues were lung, breast, colon, liver, and prostate; for hpaVisPatho, the target cancers were breast, colorectal, liver, lung, and prostate cancers; and for hpaVisSubcell, the reliability levels were set to “enhanced”, “supported”, or “approved.

### 2.5. Genomic Alterations

Consulting the cBioPortal for Cancer Genomics (https://www.cbioportal.org/, accessed on 20 August 2024), we search for mutation rates of OSRG. We analyzed individually datasets for each type of cancer, selecting different cohort studies. In order to maintain the objectivity of the study, we chose to indicate the top five genes with the highest frequency of mutations for each type of cancer.

## 3. Results

### 3.1. Identification and Enrichment of Genes Associated with Oxidative Stress

Our initial query of the Gene Ontology (GO) Resource database identified 1150 genes associated with oxidative stress. After applying our identifier filters, 685 oxidative stress-related genes (OSRG) were retained. The Gene Ontology enrichment analysis categorized these genes into three main aspects: biological processes, cellular components, and molecular functions. The top five most enriched GO terms for each category are presented in Table 1.

**Figure 1:**
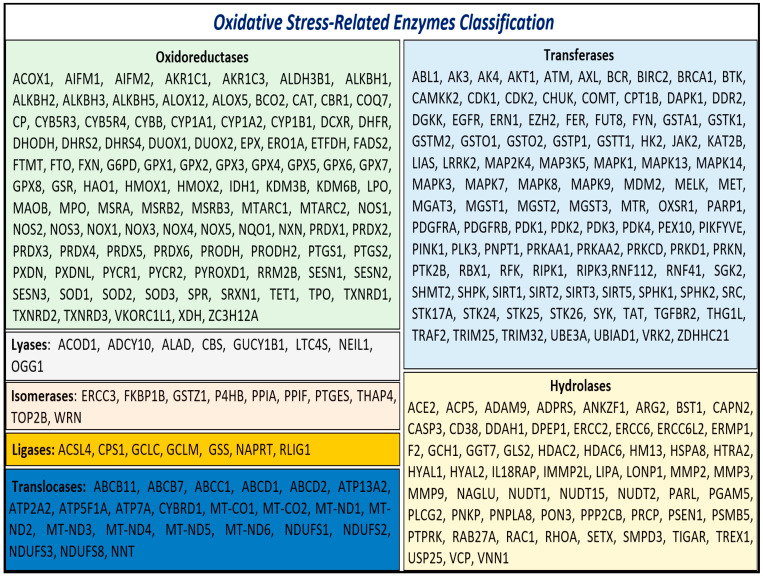
Class of OSRE that are associated with carcinogenesis events.

Given the critical role of enzymatic activity in both the generation and mitigation of oxidative stress, we focused on the enzymes identified in our analysis. These enzymes, referred to as oxidative stress-related enzymes (OSRE), totaled 313. Among these, 102 were classified as oxidoreductases, 104 as transferases, 59 as hydrolases, 8 as lyases, 10 as isomerases, 7 as ligases, and 23 as translocases (Figure 1).

### 3.2. Protein–Protein Interaction (PPI) Network and Hub Gene Identification

**Figure 2:**
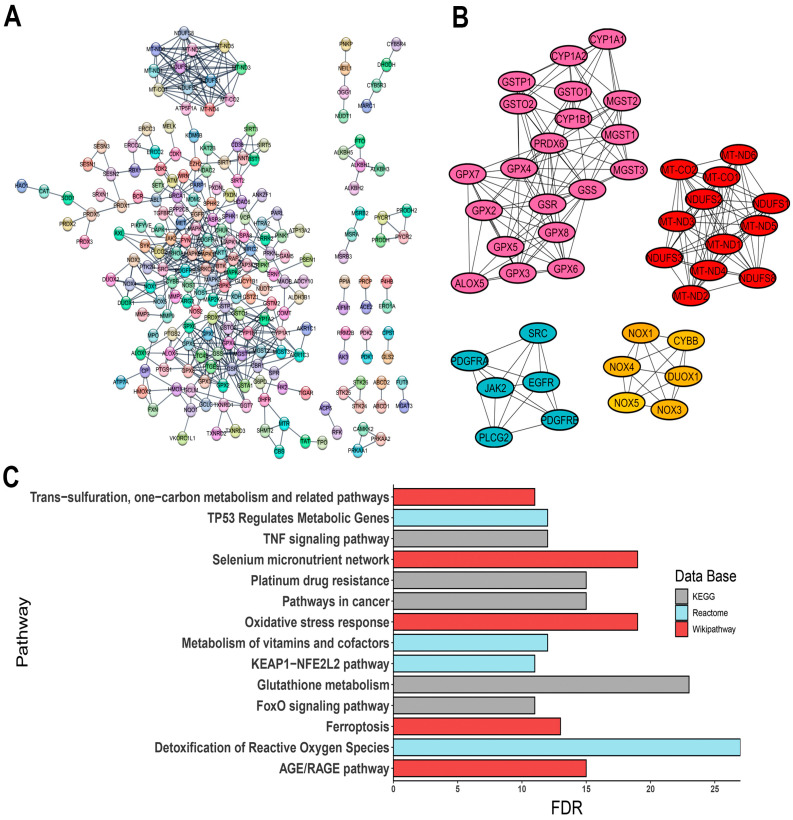
Interaction of OSRE and their enrichment pathway terms. (**A**) PPI network; (**B**) clusters grouping hub genes; (**C**) three distinct pathway enrichment results. The PPI network was constructed using STRING database (https://string-db.org/, accessed on 22 May 2024).

The constructed PPI network comprised 310 nodes with an average node degree of 21.1 and a PPI enrichment *p*-value of <1.0 × 10^−16^ (Figure 2A). PPI network was provided by STRING database and was analyzed in the Cytoscape software. Given the translational potential of our research, using a vast number of genes for clinical applications would be impractical. To narrow down the focus, we utilized the CytoHubba plugin to identify hub genes, selecting those with score values greater than 100, suggesting a high topological connectivity in the protein interaction network. As a result, 44 hub genes were identified, including:

- ALOX5, encoding 5-lipoxygenase (5-LO);
- CYBB, a component of the microbicidal oxidase system in phagocytes;
- DUOX1, encoding a dual-function oxidase;
- Several cytochrome P450 genes, such as CYP1A1, CYP1A2, and CYP1B1;
- Protein tyrosine kinase genes, including EGFR, JAK2, PDGFRA, PDGFRB, and SRC;
- Various isoforms of glutathione peroxidase: GPX2, GPX3, GPX4, GPX5, GPX6, GPX7, and GPX8;
- Genes involved in glutathione metabolism, such as GSR, GSS, GSTO1, GSTO2, GSTP1, MGST1, MGST2, and MGST3;
- PLCG2, a phospholipase tyrosine kinase;
- PRDX6, a classic antioxidant enzyme;
- ROS-generating enzymes NOX1, NOX3, NOX4, and NOX5;
- Mitochondrial respiratory complex genes, including MT-CO1, MT-CO2, MT-ND1, MT-ND2, MT-ND3, MT-ND4, MT-ND5, MT-ND6, NDUFS1, NDUFS2, NDUFS3, and NDUFS8.

The interactions of these genes are visualized in Figure 2B.

### 3.3. Enrichment Pathway Analysis

Pathway enrichment analysis revealed significant associations with pathways such as glutathione metabolism, ferroptosis, oxidative stress response, and various signaling pathways, including KEAP1-NFE2L2, AGE/RAGE, and FoxO signaling, along with cancer-related oxidative stress events (Figure 2C). Additionally, gene family analysis identified 23 annotations directly linked to oxidative stress-related events, such as NADH oxidoreductase core subunits, glutathione S-transferases, peroxiredoxins, mitogen-activated protein kinases, and selenoproteins (Figure S1, Table S1). To validate the relevance of the identified OSRE to the cancers of interest, we consulted ProteomicsDB 2020 via the Enrichr platform (https://maayanlab.cloud/Enrichr/, accessed on 6 June 2024). Our analysis demonstrated a strong association between the selected genes and the five tumor cell lines (Table 2).

### 3.4. Deregulated OSRE in Cancer Samples

**Figure 3:**
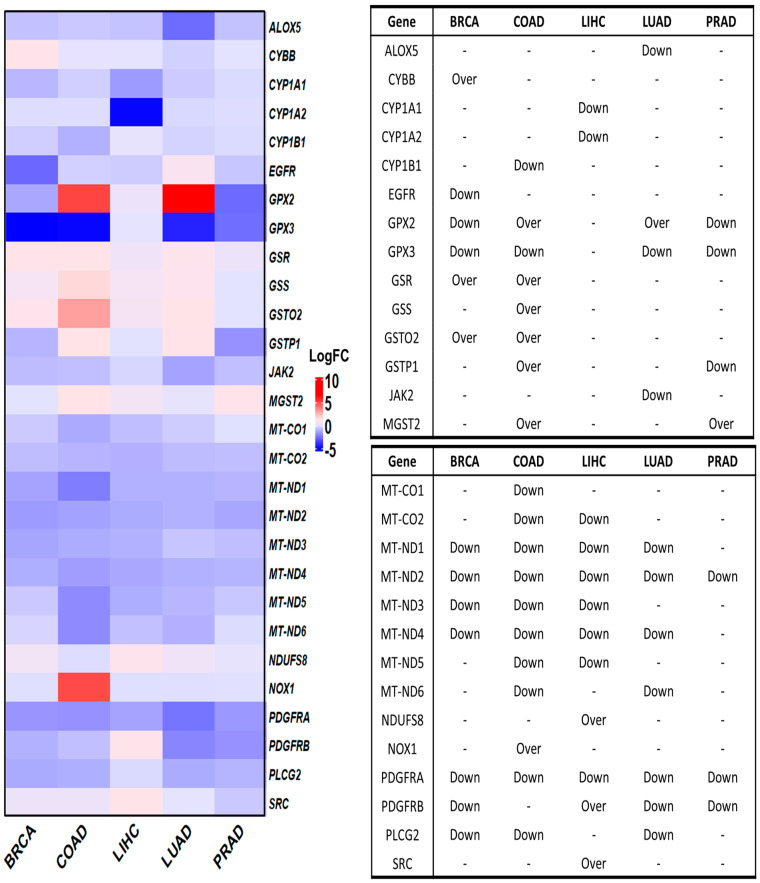
Differential gene expression of OSRE by cancer type. Left panel shows de fold change on genes by individually types of cancer, considering BRCA (num(T) = 1085; num(N) = 291), COAD (num(T) = 275; num(N) = 349), LIHC (num(T) = 369; num(N) = 160), LUAD (num(T) = 483; num(N) = 347), and PRAD (num(T) = 492; num(N) = 152). The statistically significant differentially expressed genes are indicated in the panel to the right. Over means that the gene is overexpressed in tumor samples (T) compared to normal samples (N). Breast Cancer (BRCA), Colon Adenocarcinoma (COAD), Liver Hepatocellular Carcinoma (LIHC), Lung Adenocarcinoma (LUAD), and Prostate Cancer (PRAD). Data retrieved from GEPIA2 (http://gepia2.cancer-pku.cn/, accessed on 12 June 2024).

We initially investigated the differential expression of OSRE genes in tumor samples using the GEPIA database. Of the 44 identified OSRE genes, 16 showed no differential expression in breast, colorectal, liver, lung, and prostate cancers. Only MT-ND2 and PDGFRA were consistently downregulated across all five cancer types. However, several genes were uniquely deregulated in specific tumors: ALOX5 and JAK2 in LUAD, CYBB in BRCA, CYP1A1, CYP1A2, NDUFS8, and SRC in LIHC, CYP1B1, and GSS in COAD. Interestingly, three genes exhibited contrasting expression patterns in different cancer types. For instance, GPX2 was overexpressed in colon and lung cancers but downregulated in breast and prostate cancers. Similarly, GSTP1 was overexpressed in colon cancer but downregulated in prostate cancer, while PDGFRB showed an overexpression in liver cancer and downregulation in lung cancer (Figure 3).

We also examined the mutation rates of OSRE genes using cBioPortal. For colorectal cancer, we found altered genes in 1218 (23%) of 5280 patients, while in breast cancer, mutations were observed in 2341 (30%) of 7908 patients. Liver cancer showed altered OSRE in 670 (12%) of 5480 patients, lung cancer in 2547 (32%) of 7956 patients, and prostate cancer in 1730 (14%) of 12,163 queried patients.

**Figure 4:**
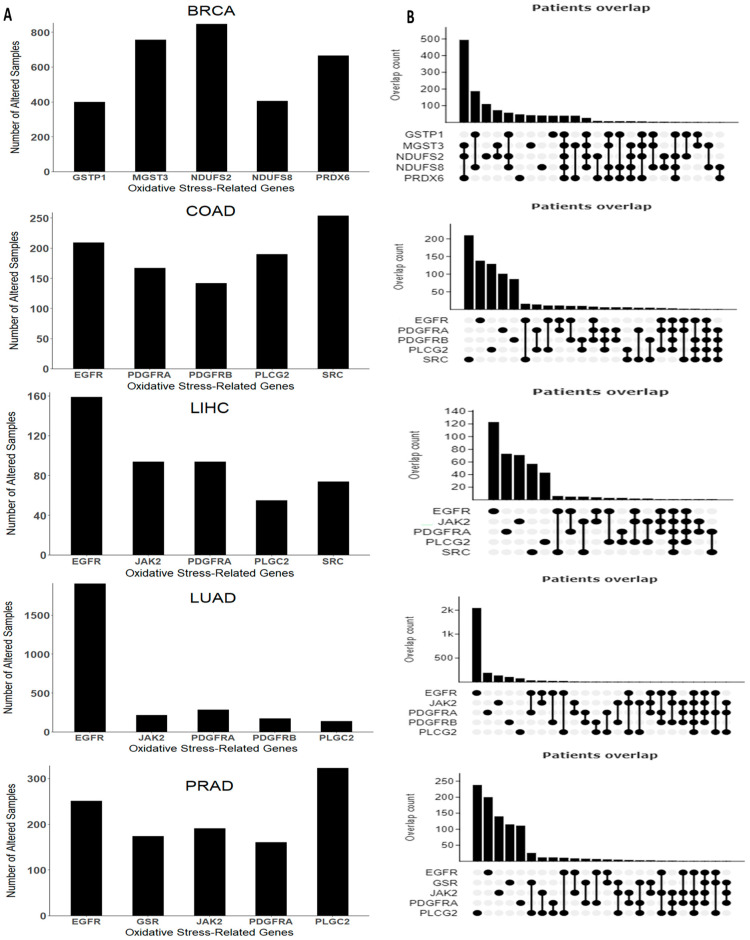
Genomic alterations arranged by cancer type. (**A**) Top five genes with higher frequency and (**B**) co-expression pattern of the top five genes. Breast Cancer (BRCA), Colon Adenocarcinoma (COAD), Liver Hepatocellular Carcinoma (LIHC), Lung Adenocarcinoma (LUAD), and Prostate Cancer (PRAD). Data retrieved and adapted from cBioPortal for Cancer Genomics (https://www.cbioportal.org/, accessed on 20 August 2024).

The top five mutated OSRE genes and their co-expression patterns are depicted in Figure 4. Notably, BRCA patients showed a higher frequency of simultaneous mutations in MGST3, NDUFS2, and PRDX6, while co-occurring mutations were less frequent in other tumor types. Unique mutations in genes such as NDUFS2, MGST3, PRDX6, NDUFS8, and GSTP1 were found predominantly in breast cancer. However, some OSRE genes, like JAK2 and PLCG2, were shared across liver, lung, and prostate cancers. Additionally, EGFR and PDGFRA mutations were observed in liver, lung, colorectal, and prostate cancers. The most frequent type of genetic alterations are amplifications, especially in BRCA, and deep deletions, mainly in PRAD. Other types of genetic alterations were missense and inframe mutations. The heterogeneity of the alterations may be a critical factor in both the biology of the tumor and the clinical outcome. The distribution of structural alterations in each cancer type is shown in Figure S2, and genetic alterations are depicted in Figure S3.

### 3.5. Upstream Regulators of OSRE Genes

**Figure 5:**
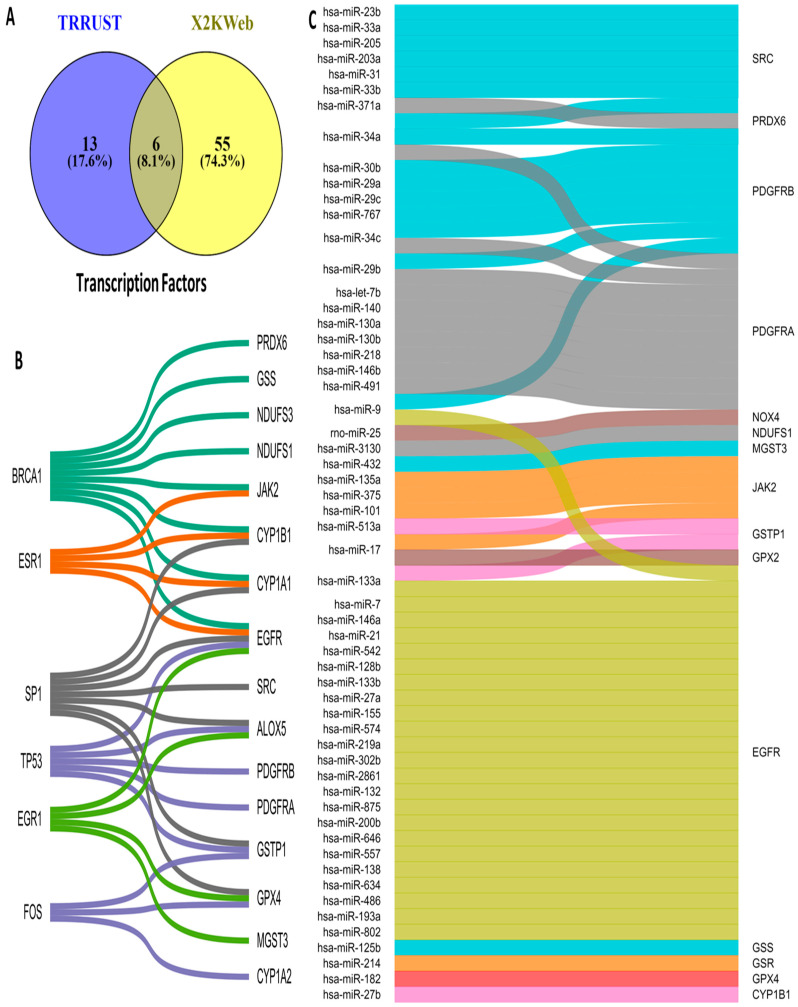
Upstream regulators of OSRE. (**A**) Venn diagram illustrating the number of OSRE’ transcription factors found in two distinct databases, (**B**) TFs and their candidate regulate-genes, and (**C**) miRNAs and their candidate-regulated genes.

The regulation of OSRE gene expression is controlled by transcription factors (TFs) and microRNAs (miRNAs). To identify potential upstream regulators of OSRE genes, we consulted two different databases: TRRUST and X2KWeb. Our analysis revealed 74 possible TFs, 19 from TRRUST and 61 from X2KWeb. Interestingly, only six TFs were common across both databases: BRCA1, ESR1, SP1, TP53, EGR1, and FOS (Figure 5A). The number of OSRE genes regulated by these TFs is as follows: BRCA1 regulates eight OSREs, SP1 regulates seven, TP53 regulates five, EGR1 and ESR1 regulate four each, and FOS regulates three genes. Notably, EGFR is regulated by five different TFs, while CYP1A1, CYP1B1, ALOX5, GSTP1, and GPX4 are each regulated by three distinct transcription factors (Figure 5B).

Regarding miRNA regulation, we identified a broad diversity in how OSRE genes are controlled. We found that 62 miRNAs may regulate 17 OSRE genes. Among these, EGFR stands out, being regulated by 25 different miRNAs. PDGFRA and PDGFRB are regulated by nine miRNAs each, SRC by seven, JAK2 by six, MT-CO2 by five, while NOX4, CYBB, GSTP1, and CYP1B1 are each regulated by three miRNAs. Additionally, ALOX5 and PRDX6 are regulated by two miRNAs each. Two miRNAs were of particular interest: hsa-miR-34a, which regulates PDGFRA, PDGFRB, and SRC, and hsa-miR-1, which controls the expression of EGFR, MT-CO1, and MT-ND1. Several other miRNAs (hsa-miR-24a, -9, -29b, -34c, -17, -101, -146b, -204, -218, -137, -164a, -125b, -135a, -133a, -133b, and -27b) were found to regulate two distinct OSRE genes (Figure 5C). These findings highlight the complexity of oxidative stress regulation, revealing a highly intricate network of TFs, miRNAs, and OSRE genes that orchestrate cellular responses to oxidative stress.

### 3.6. Protein Expression of OSRE

Gene expression does not always directly correlate with protein levels, making it essential to evaluate protein expression patterns in both normal and cancerous tissues. Using the Human Protein Atlas (HPA) platform, we analyzed the protein expression of OSRE hub genes. Our analysis showed that several OSRE proteins, such as MGST2, MT-CO1, MT-CO2, MT-ND3, NDUFS2, PLCG2, and PRDX6, exhibit medium to high expression in various normal tissues (Figure S4A). However, the HPA platform did not return results for certain OSRE genes, including GPX5, GPX6, GPX7, GPX8, MT-ND2, MT-ND5, MT-ND6, and any members of the NOX gene group.

**Figure 6:**
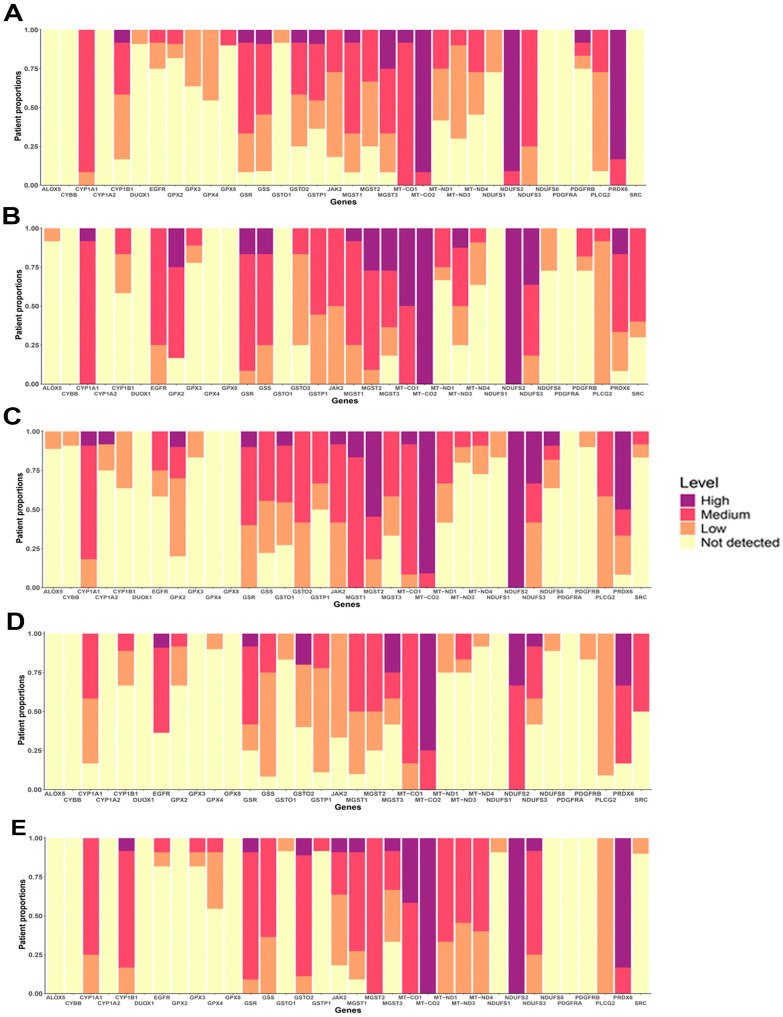
Protein expression of OSRE in (**A**) Breast cancer, (**B**) Colorectal cancer, (**C**) Lung cancer, (**D**) Liver hepatocelular cancer and (**E**) Prostate cancer.

In terms of subcellular localization, enzymes encoded by the hub genes were predominantly found in mitochondria, cytoplasm, and nucleoplasm compartments (Figure S4B). In samples of patient with breast, colorectal, liver, lung, and prostate cancers, OSRE showed medium to high expression levels, further highlighting their potential importance in tumorigenesis, cancer progression, and patient survival (Figure 6). Just to illustrate the differences in protein expression between tumor and normal tissues, we generated a histopathological atlas comparing MGST1 immunohistochemistry results in both tissue types (Figure S5). This comparative analysis highlights significant variations in protein expression patterns, reinforcing the potential role of OSRE in the carcinogenic process.

## 4. Discussion

Enzymes play dual roles in OS, functioning as both antioxidants and prooxidants, depending on cellular conditions. Classic antioxidant enzymes such as SOD, catalase, and GPx neutralize ROS, protecting cells from oxidative damage. However, when antioxidant defenses are overwhelmed or malfunction, enzymes like NADPH oxidase, mitochondrial respiratory complexes, and intermediary metabolism enzymes can become prooxidants, contributing to elevated OS. This imbalance can drive various pathological conditions, including cancer. The interplay between oxidant generation and antioxidant defense is more intricate than the classical triad of SOD, catalase, and GPx, necessitating precise regulation of numerous enzymes to maintain redox homeostasis.

Our study identified specific genes coding OSREs that are associated with cancer. Among the molecular functions enriched, oxidoreductase activity stood out, suggesting that class 1 enzymes are central to the carcinogenesis process. In fact, we identified a large proportion of enzymes classified under oxidoreductases (E.C. 1), which are crucial in processes ranging from energy metabolism to detoxification and signaling—functions frequently deregulated in cancer [7]. Key examples of oxidoreductases found in our analysis include, beyond catalase, SOD and GPx, NADPH oxidase (NOX), thioredoxin reductase (TXNRD), and cytochrome P450 (CYP) isoforms. These enzymes demonstrate how OS management intersects with tumor biology. Transferases (E.C. 2) were the most prevalent class of enzymes in our findings. These enzymes are integral to cellular detoxification, often modulating OS by catalyzing the conjugation of ROS and other harmful metabolites with glutathione or acetyl groups. Glutathione S-transferase (GST) isoforms, including microsomal GSTs (MGST), were prominently involved in this process. Genes encoding translocases (E.C. 7), including respiratory chain complex proteins such as cytochrome c oxidase (MT-CO) and NADH dehydrogenase iron-sulfur protein (NDUFS), were also significantly enriched. Mitochondrial disruption, particularly in the ETC and OXPHOS, contributes to OS, mitochondrial dysfunction, genomic instability, and metabolic reprogramming—all hallmarks of cancer [27,28].

Our analysis of key cancer types revealed differential expression of genes encoding NADH-ubiquinone oxidoreductase and platelet-derived growth factor receptor alpha (PDGFRA). In most tumors, except prostate cancer, genes MT-ND1 through MT-ND4, which are crucial for the assembly of ETC Complex I, showed reduced expression. This finding highlights the role of mitochondrial genome deregulation in cancer progression, response to therapy, and prognosis [29]. Approximately 50% of cancers harbor somatic mutations in mtDNA, making it one of the most frequently mutated regions in cancer genomes [27].

PDGFRA downregulation was observed across the five tumor types analyzed. This gene encodes a receptor tyrosine kinase critical for cellular growth, angiogenesis, and survival via activation of the PI3K/AKT, RAS/MAPK, and STAT pathways [30,31]. Alterations in PDGFRA, including mutations and amplifications, are frequently found in colorectal, liver, lung, and prostate cancers. The PDGFR ligand, platelet-derived growth factor (PDGF) has its expression strongly correlated with distinct types of tumors [32]. PDGF signaling can enhance ROS production, inactivating MAP kinase phosphatases and increasing tumor proliferation and progression [33]. Based on our results, we suggest that the oncogenic role of PDGFR may be associated with gene amplification rather than regulation of its expression. Genetic alterations like gene amplification and translocation can modulate PDGFR signaling pathways, such as Notch, Matrix metalloprotein and TGFβ signaling, favoring oncogenic responses like epithelial mesenchymal transition, angiogenesis, migration, and metastasis [34].

Alterations in ETC Complex I are evident in multiple tumor types. Mutations in MT-ND1 account for nearly 40% of mtDNA mutations in colorectal cancer, correlate with breast cancer, and promote lung tumorigenesis. Inhibition of Complex I may reduce drug resistance and metastatic progression in prostate cancer. Likewise, heteroplasmic mutations in MT-ND1 are linked to hepatocellular carcinoma [35,36]. Additional MT-ND mutations have been reported across BRCA, COAD, PRAD and LIHC, further supporting the importance of mitochondrial function in tumor biology [37,38,39].

Several genes involved in the glutathione system exhibited altered expression patterns in cancer. GPX3 was downregulated in breast, colorectal, lung, and prostate cancers, with GPX2 showing a similar pattern in all but colorectal and prostate cancers. GPX3 functions as a tumor suppressor, modulating apoptosis and cell growth; its downregulation compromises cellular metabolism, favoring tumor survival [40]. The differential role of GPXs across cell types and compartments highlights their importance in maintaining redox balance in cancer. This may not be unexpected, as all cells are subjected to OS, and by converting GSH to GSSG, glutathione peroxidases prevent and/or minimize the damage to cell’s ultrastructure. Additionally, GPX3 may reduce autophagy and apoptosis through the mTOR and caspase-3 pathways, minimize endoplasmatic reticulum (ER) stress while modulating pro-inflammatory responses via NF-κB/TNF-α [40]. A causal relationship between GPX3 and NOX has already been pointed out in the development of chronic kidney disease. Li, et al. [41] evidenced that reduced GPX3 expression triggers NOX4 mRNA and protein expression, culminating in increased OS. For COAD, similar relationship may occur, as we found down and over regulation of GPX3 and NOX1, respectively.

The cytosolic and mitochondrial GSH/GSSG pool is crucial for maintaining the cellular redox status, but cells can sustain the highly reduced intracellular status even in the absence of GSR. An alternative pathway to GSH synthesis may involve the NADPH-TrxR1 system [42]. Glutathione reductase (GSR) was overexpressed in breast and colorectal cancers, emphasizing the role of the glutathione system in tumor survival. Protein expression data corroborate this finding, as in the five types of cancer, GSR expression was detected within medium to high levels. In contrast, other glutathione-related enzymes, such as GSTO1 (Glutathione S-Transferase Omega 1) and GSTP1 (Glutathione S-Transferase Pi 1), exhibited distinct expression patterns across different cancers, pointing to complex regulatory mechanisms at play. The involvement of these enzymes in detoxification, particularly in liver tissues, suggests a crucial adaptive response to oxidative stress during carcinogenesis. GSTO2 polymorphisms were found in patients with prostate cancer [43], and we showed medium to high levels of GSTO2 in PRAD samples. Microsomal Glutathione S-Transferase 2 (MGST2) is involved in the production of mediators of inflammation and is associated with enhanced oxidative and ER stress, ferroptosis and apoptosis [44]. MGST2 was significantly downregulated after the treatment with a nanozymatic system in CT26 (colon carcinoma) and MKN45 (human gastric adenocarcinoma) cells, as well as in a tumor-bearing mice model [45]. The result we found for GSH-related enzymes, points the role of those proteins to assist in the adaptation to oxidative stress that happens during the carcinogenesis process. These findings warrant further investigation into their potential as therapeutic targets.

Using the CBioPOrtal database, we sought to identify which hub genes have the highest mutation rates in each tumor type. Our analysis revealed that mutations in OSRE such as MGST3, GSTP1, NDUFS2, NDUFS8 and PRDX6 were particularly prevalent in breast cancer, and for the five genes the most prevalent genetic alteration was amplification. The PRDX protein family is closely linked to the level of malignancy, recurrence, and prognosis in various types of cancers [46]. PRDX6, which has been associated with tumor progression, is primarily found in the cytoplasm, as we demonstrated, but it can move to compromised mitochondrial membranes to reduce ROS levels [47]. Recently, PRDX6 was proposed as a potential BRCA biomarker [48]. Moreover, EGFR mutations were frequent in colorectal, lung, prostate, and liver cancers. Osimertinib, a EGFR tyrosine kinase inhibitor, is the standard treatment for non-small cell lung cancer patients with EGFR mutations. However, approximately 10% of EGFR mutations in LUAD involve exon 20 insertions, which do not respond to standard EGFR tyrosine kinase inhibitors [49]. Elevated EGFR expression is associated with a higher risk of high-grade, advanced PRAD and prostate-specific antigen recurrence. The phosphorylation of EGFR by Traf2- and Nck-interacting kinase (TNIK) is critical for its intracellular localization and transcriptional activity. The extracellular domain (ECD) of EGFR can bind specific ligands, facilitating EGFR activation. Inhibiting the TNIK/EGFR axis has shown potential therapeutic effects in various tumors, including COAD and LUAD. TNIK not only phosphorylates EGFR but also regulates the Wnt/β-catenin signaling pathway, increases Rho GTPase activating protein 29 (ARHGAP29) activity, promoting epithelial-to-mesenchymal transition (EMT) and cell invasion. Additionally, TNIK plays a role in ferroptosis, which is closely linked to ROS generation [50,51].

Our findings indicate that the phosphatidylinositol-specific phospholipase Cγ2 (PLCG2) gene and its protein expression are reduced in LUAD, COAD, LIHC, and PRAD. PLCG2 is frequently mutated in these tumors. Overexpression of PLCG2 reduces LUAD cell proliferation and inhibits the growth of colorectal xenografts cancer in vivo [52,53]. PLCG2 is a transmembrane signaling enzyme involved in inflammation, immunity-related diseases, and cancer [54]. Upon activation, PLCG2 cleaves phosphatidylinositol-4,5-bisphosphate (PIP2) into diacylglycerol (DAG) and inositol 1,4,5-trisphosphate (IP3), second messengers that are required to control a myriad of cellular events, including proliferation, endocytosis, and Ca^2+^ metabolism. The pro-tumorigenic role of PLCG2 may be linked to increased ROS production. Activation of PLCG2, ERK-2, and MAPK enhances NADPH oxidase activity and H_2_O_2_ production [55].

Two other enzymes that are frequently mutated in LIHC and COAD, and in LIHC, LUAD, and PRAD were SRC and JAK2, respectively. SRC (SRC Proto-Oncogene) is a non-receptor tyrosine kinase that activates downstream mediators, promoting vascular cell differentiation, proliferation, migration, and cytoskeletal reorganization. SRC is a marker of cellular invasion and metastasis. SRC can contribute to ROS generation by activating NADPH oxidases, and OS can also modulate SRC activity [56]. Phosphorylation of SRC is inhibited by antibiotics such as tigecycline and tetracycline, which reduces SRC phosphorylation in human COAD cell lines (HT29, HCT15, HCT116, and Colo205), with a more pronounced effect in high-metastatic cells like HCT116 and Colo205. These antibiotics may also inhibit mitochondrial biogenesis and function, specifically respiratory Complex-I assembly and activity, leading to increased OS and ATP depletion [57]. In LIHC cells (HuH6 and Huh-7), inhibition of the prostaglandin E2 (PGE2) receptor EP1 reduces cell invasion and viability. PGE2-induced transactivation of EGFR in HCC cells occurs through activation of c-Src, followed by c-Met activation [58]. The JAK2/STAT3 signaling pathway upregulates the expression of Bcl2, cyclin D1, c-Myc, and other proteins linked to progression, drug resistance, and poor prognosis in COAD, LUAD, and BRCA [59]. By studying human non-small-cell lung cancer cell lines, HCI-H1299, A549, HCI-H1373, HCI-H460 and HCI-H1573, Lu et al. [59] showed the overexpression of PSMA-5, an α5 subunit of the 20S core proteasome involved with protein degradation. Through PSMA5-mediated mechanism, the inhibition of JAK2/STAT3 pathway led cell to apoptosis and sensitized LUAD cells to cisplatin. Also, activation of JAK2 pathway exerted oncogenic effects on HCC cells [60]. Oxidative stress is linked with PSMA-5 as Nrf2 signaling and proteasomal dysfunction may be associated with carcinogenesis. ROS generated by NOX4, mediated Nrf2/ARE signaling activation, causing up-regulation of PSMA5 expression [61]. In colon cancer, increased Nrf2 activity correlates with enhanced proteasome activity [62]. Although Nrf2 is recognized as a master regulator of OSRE, its role in carcinogenesis requires further investigation, as Nrf2 and its downstream genes are often overexpressed in various cancer cell lines and tissues [63]. Despite the dual relationship between OS and the JAK2/STAT3 pathway [64], JAK2 has a critical role in the mediation of tumorigenesis.

Finally, we showed that OSRGs are regulated by different transcription factors and miRNAs. Although we will not go into detail, it is possible to observe that, in addition to Nrf2, other factors such as BRCA1, ESR, SP1, TP53, EGFR1, and FOS can play an important role during the carcinogenesis process. The SRC, PDGFRA, PDGFRB, and EGF genes also appear to be under the regulation of a large number of miRNAs. The importance of regulating oncogenes and tumor suppressors by mechanisms involving miRNAs has already been well established and demonstrated in different studies [65]. Just to give an idea, using the Pubmed database and selecting the terms “miRNA” AND “cancer”, more than 18,000 articles have been deposited in the last two years alone (2022–2024). In this same scenario, the relationship between miRNAs and OS is also well established for different conditions, including cancer [66,67,68].

## 5. Conclusions

Our study brought non-classical enzymes that may be of relevance for carcinogenesis, revealing that these enzymes, beyond the traditionally studied ones, are crucial in tumor development and progression. While much of the research has focused on well-established oncogenic pathways and classic enzymes, our findings highlight the importance of less conventional players, such as PDGFRA, GSTs, ETC complex I, PLCG2, PRDX6, SRC, and JAK2, at least in the most prevalent types of cancers including BRCA, COAD, LUAD, PRAD, and LIHC. In this scenario, we point to a broader class of enzymes that significantly influence tumorigenesis, suggesting that therapeutic strategies targeting these non-classical enzymes could offer new avenues for cancer treatment. Further exploration of these enzymes, at gene or protein level, may be useful for cancer treatment, particularly by modulating redox homeostasis and targeting mitochondrial vulnerabilities.

## 6. Limitations

We would like to comment on some of the limitations of our approach. First, while our study identified a range of OSRE beyond the classical ones that play a role in carcinogenesis, much of the data is derived from in vitro and preclinical models. This may limit the generalizability of our findings to clinical settings. Second, the reliance on publicly available databases and bioinformatic tools, while powerful, introduces potential biases related to data accuracy, annotation quality, and sample heterogeneity. Additionally, our focus on OSRGs and their regulatory pathways provides valuable insights, but it does not capture the full spectrum of other contributing factors in cancer biology. Finally, while we brought some potential therapeutic implications, the functional validation of enzyme interactions and their potential as therapeutic targets require further experimental studies.

## Figures and Tables

**Table 1:** Top 5 Gene Ontology results for the 1150 OSRG ranked by FDR.

GO	GO ID	GO Description	FDR
BP	GO:0034599	Response to oxidative stress	2.14^−184^
BP	GO:0062197	Cellular response to chemical stress	1.33^−98^
BP	GO:1901700	Metabolic process	7.11^−82^
BP	GO:0000302	Response to reactive oxygen species	8.51^−80^
BP	GO:0010035	Response to oxygen-containing compound	1.12^−73^
MF	GO:0016491	Catalytic activity	2.67^−153^
MF	GO:0016209	Oxidoreductase activity	2.54^−88^
MF	GO:0003824	Antioxidant activity	3.52^−50^
MF	GO:0004601	Small molecule binding	1.96^−47^
MF	GO:0019899	Peroxidase activity	9.80^−41^
CC	GO:0005739	Mitochondrion	1.65^−46^
CC	GO:0005737	Cytoplasm	1.76^−32^
CC	GO:0043227	Membrane-bound organelle	1.20^−25^
CC	GO:0005740	Mitochondrial envelope	7.96^−18^
CC	GO:0031967	Oxidoreductase complex	1.07^−15^

BP: Biological process; MF: molecular function; CC: cellular compartment; FDR: false discovery rate. Data obtained from STRING online tool.

**Table 2:** ProteomicDB results showing the significance association of OSRE with liver, prostate, lung, colorectal and breast cancer cells.

Term	*p* Value	Odds Ratio	Combined Score
Hepatoma HepG2	2.98 × 10^−8^	13.38	231.97
Prostate LNCaP	3.73 × 10^−7^	10.07	149.14
Lung A-549	1.45 × 10^−4^	11.24	99.36
Colon RKO	0.001	7.11	48.54
Breast MDA-MB	0.004	6.36	33.84

## Data Availability

The data utilized to elaborate our results are available in the Gene Ontology Resource database (https://geneontology.org/ (accessed on 15 April 2024)), Molecular Signatures Database (MSigDB, https://www.gsea-msigdb.org/gsea/msigdb/ (accessed on 15 April 2024)), ProteomicsDB (https://www.proteomicsdb.org/ (accessed on 3 June 2024)), STRING database (http://string-db.org; (accessed on 22 May 2024)), ToppGene database (https://toppgene.cchmc.org/enrichment.jsp; (accessed on 6 June 2024)), Gene Expression Profiling Interactive Analysis (GEPIA2, http://gepia2.cancer-pku.cn/ (accessed on 12 June 2024)), X2Kweb (https://maayanlab.cloud/X2K/ (accessed on 10 July 2024)), TRRUST (https://www.grnpedia.org/trrust/ (accessed on 10 July 2024)), Human Protein Atlas (HPA, https://www.proteinatlas.org/ (accessed on 5 August 2024)), cBioPortal for Cancer Genomics (https://www.cbioportal.org/ (accessed on 20 August 2024)). Other data were generated by R studio platform and specific libraries. All data are included in this published article and its Supplementary Information Files.

## Supplementary Materials

The following supporting information can be downloaded at: https://www.mdpi.com/article/10.3390/antiox13111279/s1, Figure S1: Gene family analysis annotations; Figure S2: Distribution of structural alterations in each cancer type; Figure S3: Genetic alterations found in the top 5 OSRE. Data retrieved from the cBioPortal (https://www.cbioportal.org/); Figure S4: Expression pattern of OSRE in (A) normal cells and tissues and (B) their subcellular location; Figure S5: A representative histopathological atlas comparing the expression of MGST1 in different types of tumor cells. Table S1: TOPPFUN gene family analysis.
