# Supplementary material for: In Silico Analysis of Non-Conventional Oxidative Stress-Related Enzymes and Their Potential Relationship with Carcinogenesis
Source: Antioxidants (Basel). 2024 Oct 23;13(11):1279. doi: 10.3390/antiox13111279 (PMC11591236; doi:10.3390/antiox13111279)
Supplement: Supplementary file 1 [file antioxidants-13-01279-s001.zip › antioxidants-3238767-supplementary.pdf]

SUPPLEMENARY MATERIAL

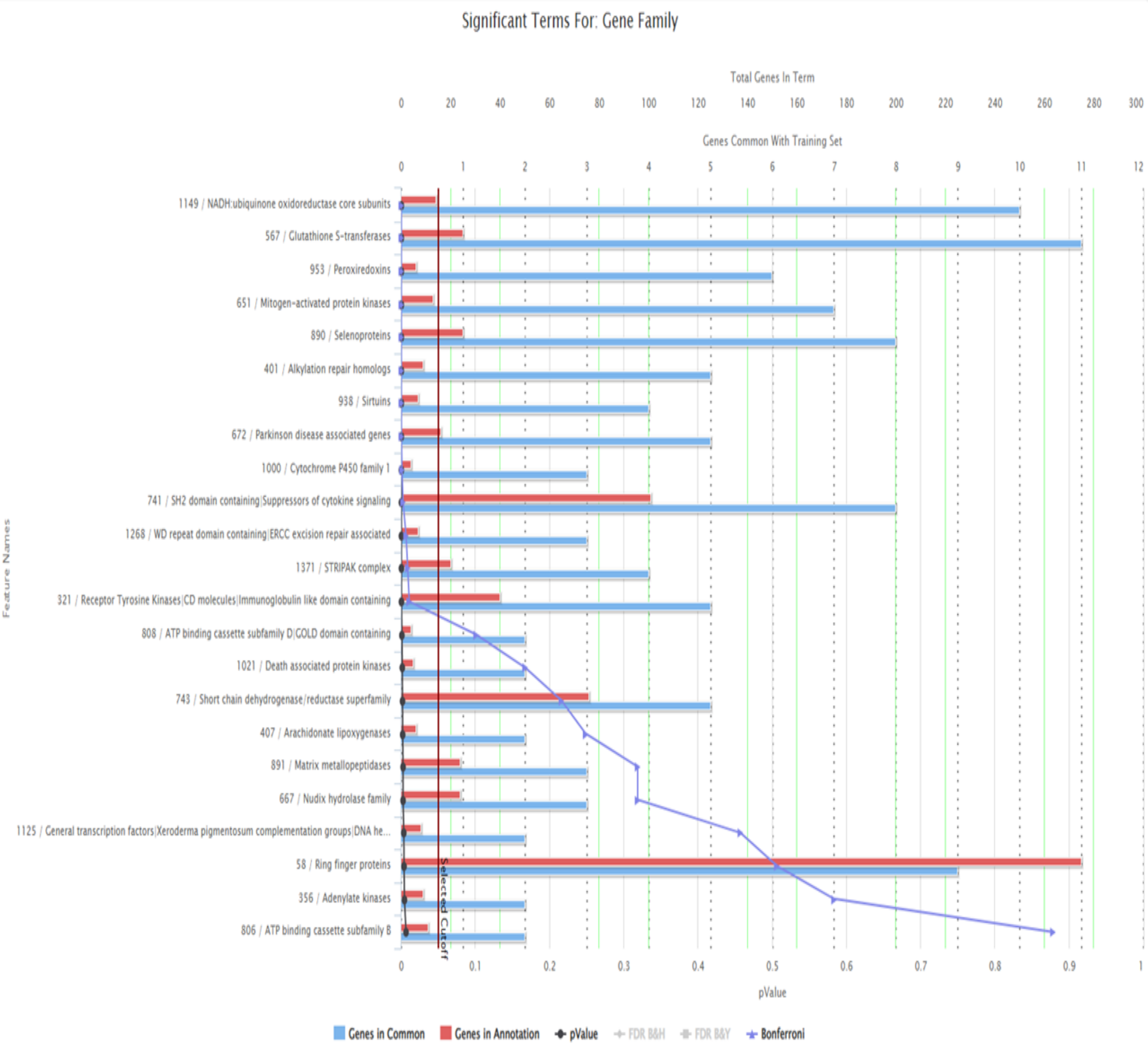

Figure S1 - Gene family analysis annotations.

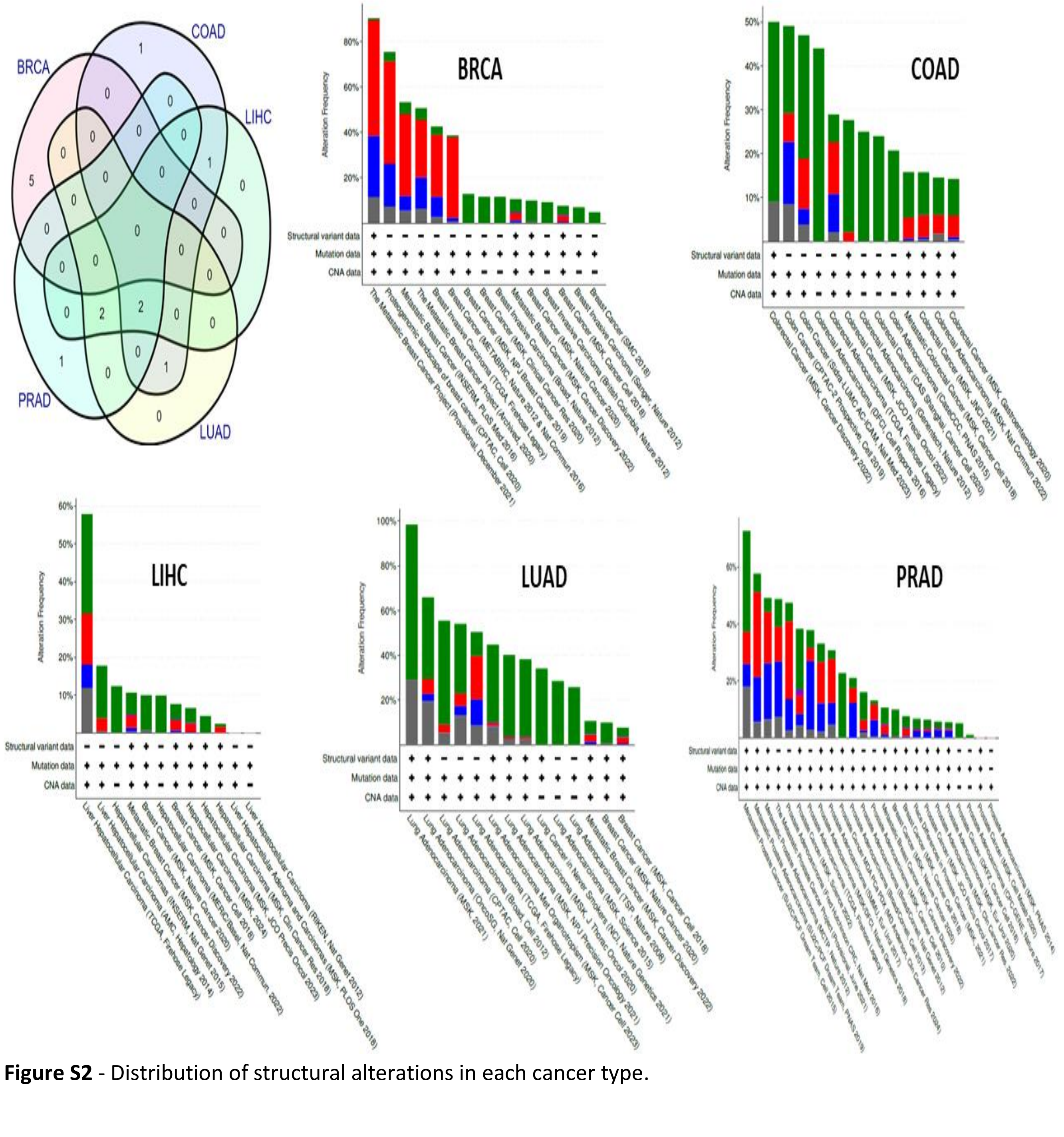

Figure S2 - Distribution of structural alterations in each cancer type.

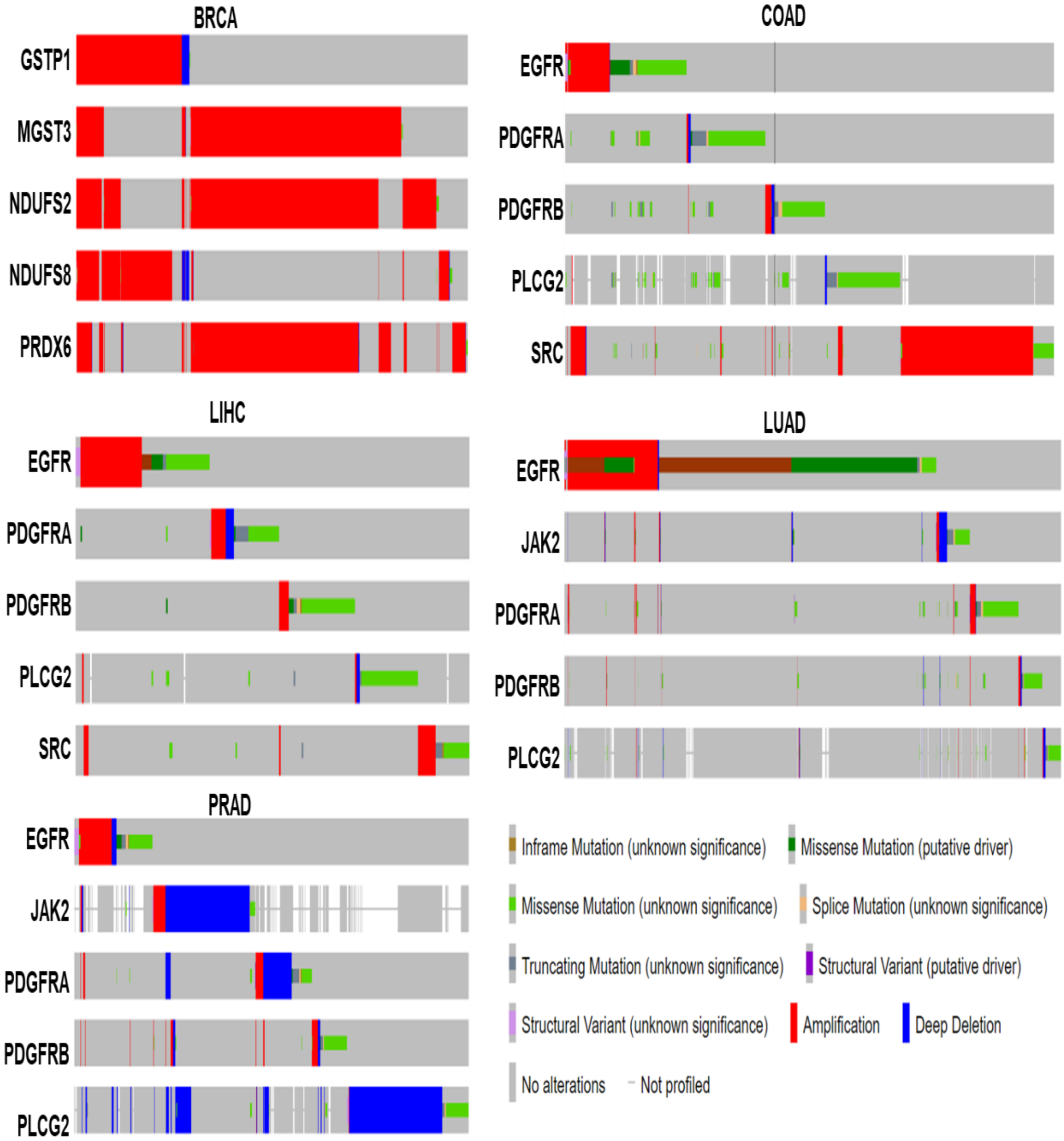

Figure S3 – Genetic alterations found in the top 5 OSRE. Data retrieved from the cBioPortal. (https://www.cbioportal.org/)

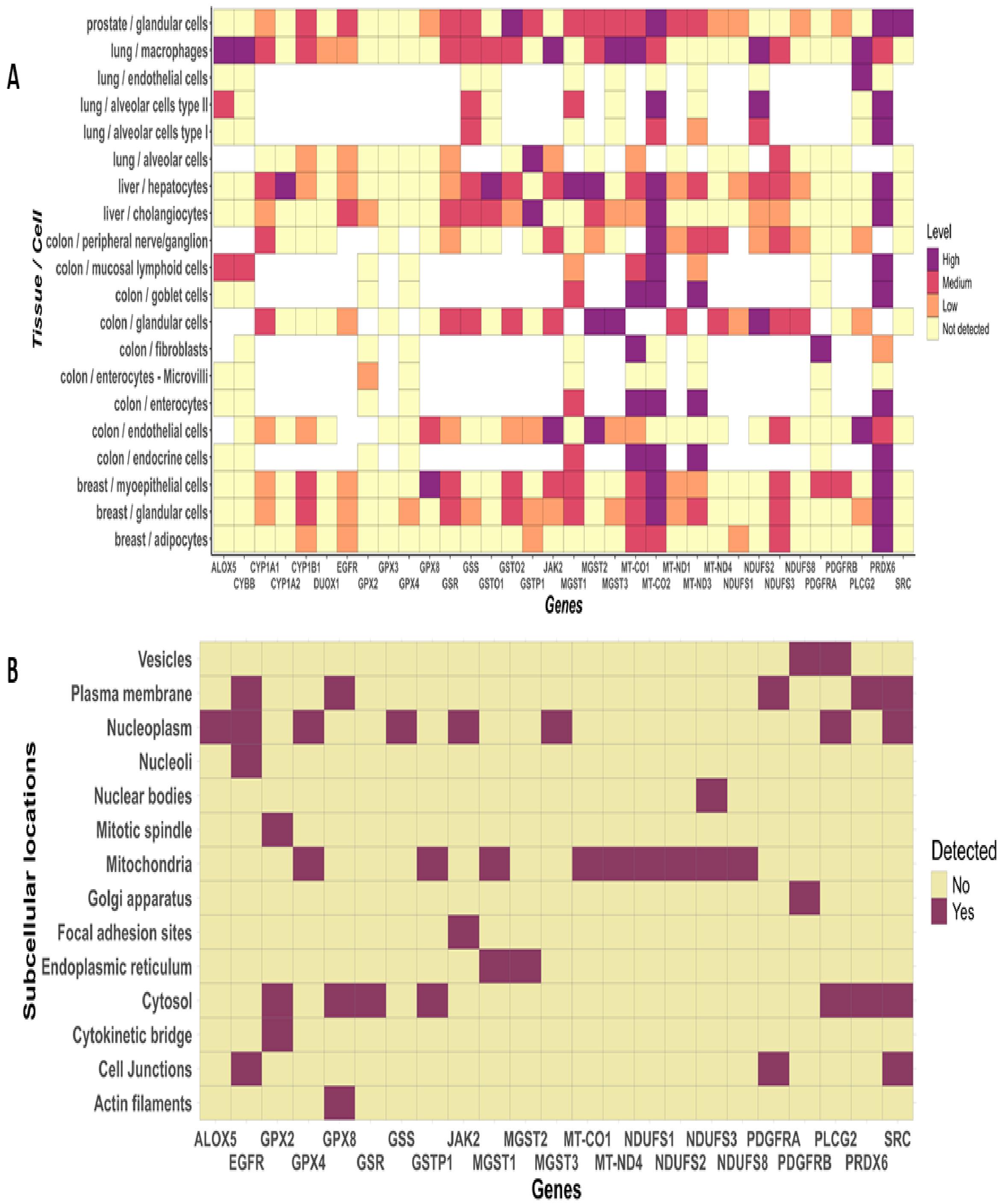

Figure S4 – Expression pattern of OSRE in **A)** normal cells and tissues and **B)** their subcellular location.

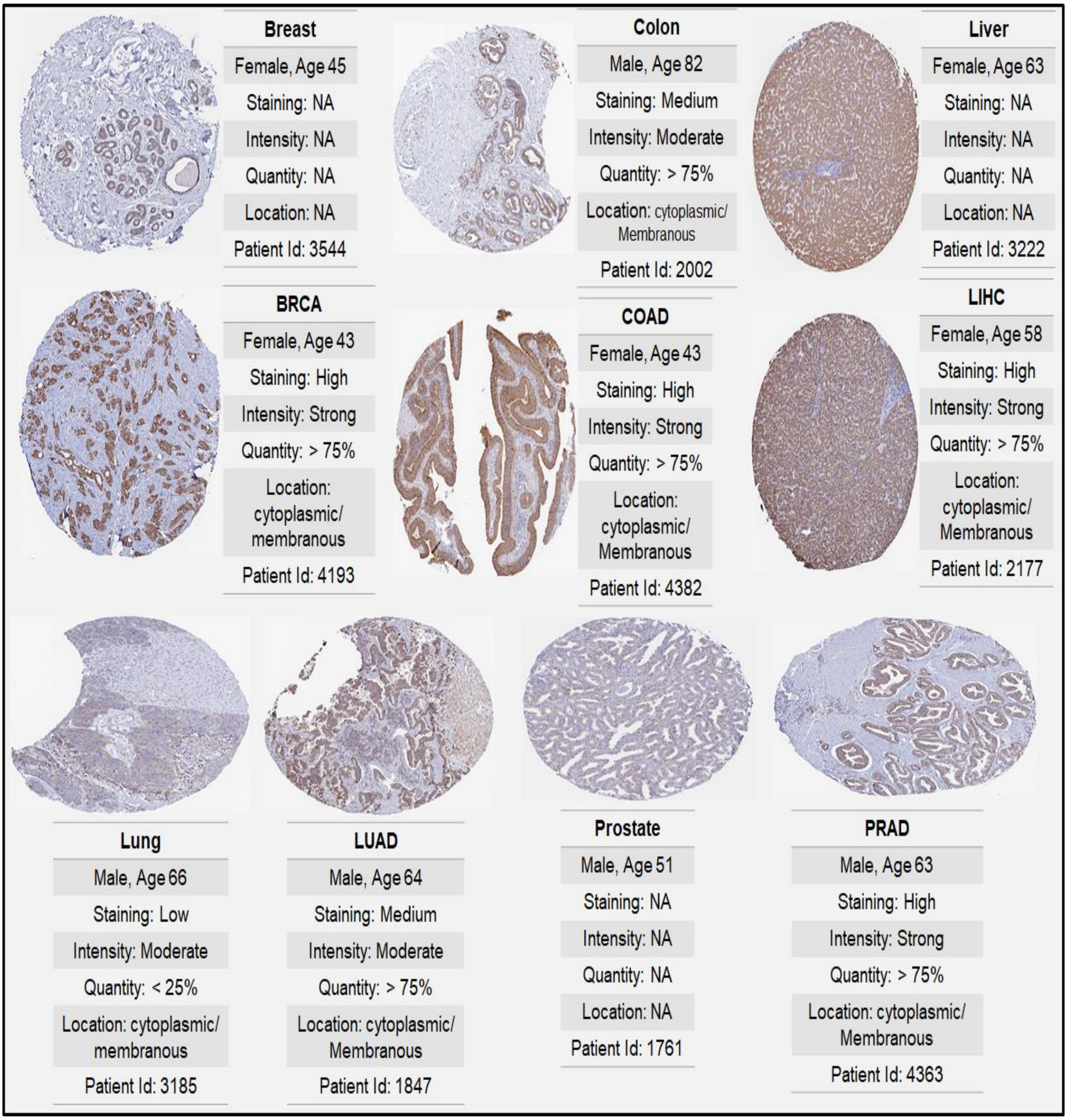

Figure S5 – A representative histopathological atlas comparing the expression of MGST1 in different types of tumor cells.
